# Recent advances in understanding multiple myeloma

**DOI:** 10.12688/f1000research.8777.1

**Published:** 2016-08-23

**Authors:** Binod Dhakal, Saulius Girnius, Parameswaran Hari

**Affiliations:** 1 Division of Hematology/Oncology, Medical College of Wisconsin, Milwaukee, WI, USA; 2 Division of Hematology/Oncology, University of Cincinnati, Cincinnati, OH, USA

**Keywords:** Multiple Myeloma, treatment, monoclonal antibodies, diagnosis

## Abstract

There have been major recent advancements in the understanding and management of multiple myeloma. Diagnostic criteria have been revised and former ultra-high-risk smoldering multiple myeloma is now considered multiple myeloma in need of treatment. Understanding clonal progression, evolution, and tides not only has helped elucidate the disease behavior but might help expand therapeutic choices in order to select appropriate treatment for patients. Unprecedented response rates with modern triplet induction therapies containing proteasome inhibitor and immunomodulators have made this approach standard for initial treatment. The US Food and Drug Administration approved four new drugs (two targeted antibodies and two oral agents) in 2015 in relapsed/refractory multiple myeloma and these drugs along with the other already-available drugs have now increased the choices of regimens. Even drugs without single-agent activity, such as panobinostat and elotuzumab, have an important role, especially in the proteasome inhibitor refractory setting. Recent studies done in the context of novel agent induction suggest that high-dose therapy followed by autologous transplant continues to improve response rates and progression-free survival, thus underscoring their role in transplant-eligible patients. Evolving paradigms in the treatment of multiple myeloma include newer promising immune approaches, such as adoptive cellular therapies, vaccines, or antibody-based immune manipulations. Though multiple myeloma is still considered incurable, it is clear that with the improved understanding of disease biology and clonal architecture of relapse combined with the availability of multi-targeted approaches, we are ever closer to a lasting cure or transformation into indolent and long-lasting disease courses or both.

## Introduction

Multiple myeloma (MM) is a clonal expansion of malignant plasma cells resulting in end organ damage, including lytic bone lesions, anemia, renal failure, or hypercalcemia. Both the understanding of MM and its management have evolved rapidly over recent years. Concomitant with the increasing incidence of MM to almost 30,000 patients annually in the US, mortality has trended down in the last decade, resulting in an increased prevalence of MM ^1^. In 2015, four new drugs—the first two monoclonal antibodies and two oral agents—were approved by the US Food and Drug Administration (FDA). Importantly, these drugs have improved the outcomes in relapsed and heavily pre-treated patients.

## High-risk smoldering multiple myeloma

Smoldering MM (SMM) was traditionally defined on the basis of laboratory criteria—monoclonal (M) protein level of more than 3 g/dl or a clonal marrow plasmacytosis of more than 10%—and the absence of end organ damage. The ability to identify persons with SMM who are likely to progress rapidly to symptomatic MM has led to the revision of the criteria that define treatment initiation. Ultra-high-risk SMM patients with an 80% risk of progression to MM in 2 years are now considered in the updated criteria to have MM requiring treatment ^2^. Currently, SMM is defined by the presence of serum M protein of at least 3 g/dl or 10 to 60% of clonal bone marrow plasma cells (or both) with no evidence of target organ damage, myeloma-defining events (MDEs), or amyloidosis ^2^. Patients with high-risk SMM have a 25% risk of progression to MM in 2 years, whereas those with low risk have a 5% risk per year ^3^. Multiple prognostic markers have been identified as surrogates for high-risk SMM ^4– 9^. Some of these include SMM with serum M protein of at least 3 g/dl, IgA isotype, involved/uninvolved free light chain ratio of at least 8 (but less than 100), bone marrow clonal plasma cells of 50 to 60%, t(4;14), del(17p), or 1q gain and Bence-Jones proteinuria. Management of high-risk SMM remains controversial after treatment with lenalidomide was shown to improve overall survival (OS) compared with a strategy of observation until target organ damage ^10^. It is possible that SMM represents a curable stage of disease wherein early intervention approaches with limited toxicity may be curative and delay or avoid end organ damage provided that treatment does not lead to the outgrowth of more resistant clones. When compared with symptomatic MM, high-risk SMM can achieve a deeper response, as measured by minimal residual disease (MRD), with 11 out of 12 patients developing MRD negativity ^11^. A longer follow-up is necessary to determine the duration of response, comparing it with the financial and physical burden of therapy. Thus, these treatments should be performed in clinical trials exploring the timing, the benefits, and the type of therapy in high-risk SMM.

## New criteria for multiple myeloma

The revised International Myeloma Working Group criteria for the diagnosis of MM requires, in addition to the presence of either 10% or more clonal plasma cells or a biopsy-proven plasmacytoma, one or more MDEs. MDE includes CRAB features (increased calcium level, renal dysfunction, anemia, and destructive bone lesions) and three specific biomarkers: (a) clonal plasma cells of at least 60%, (b) involved/uninvolved light chain ratio of at least 100, and (c) more than one focal lesion on magnetic resonance imaging. Each of these biomarkers was associated with an 80% risk of progression to symptomatic MM in 2 years in independent studies ^4, 7, 8^.

Staging of MM was recently updated to reflect the advances in treatment of MM as well as improved detection of high-risk disease. Revised-International Staging System (R-ISS) stage I disease includes ISS stage I with a normal lactate dehydrogenase (LDH) level and absence of high-risk cytogenetics (del(17p), t(4;14), or t(14;16)). R-ISS III includes ISS stage III with either high-risk cytogenetics or elevated LDH. R-ISS II includes all others. The 5-year OS rates were 82% in R-ISS I, 62% in R-ISS II, and 40% in R-ISS III ^12^.

## Clonal dominance

The initial event leading to malignant plasma cells occurs in the germinal center during isotype class switching and somatic hypermutation. These mutations are present in monoclonal gammopathies of unknown significance but are not sufficient for evolution to MM ^13^. The progression to MM involves additional oncogenic events, including activation of oncogenes *K-Ras*, *N-Ras*, *FAM46C*, *MYC*, and *BRAF* and loss of function of *p53*, epigenetic changes with histone methylation/deacetylation, and genomic instability ^14, 15^. No one common driver mutation has been identified in MM, although recurrent variants have been identified. Deregulation of the RAS/MAPK, NF-κB pathway, and apoptotic response are involved, but only the inability for effective apoptotic response to DNA damage has prognostic value ^15^. A second study confirmed that the presence of the aforementioned oncogenes did not influence OS or progression-free survival (PFS) ^16^. In MM, at least four distinct evolutionary patterns can be discerned between initial treatment phase and subsequent relapses. The simplest situation is no change in the clonal and sub-clonal composition between various time points in the natural course of disease, which represents re-emergence of the original clonal plasma cell population at relapses. About one-third of patients in this group have stable genomes and are typically associated with hyperploidy and favorable outcomes. The other three scenarios include “either differential clonal response or clonal tides” where in sub-clones are identified at different time points, but their relative proportions change over time or linear clonal evolution where a new (previously undetectable) sub-clone emerges later or a branching evolution pattern corresponding to the emergence of new clones with the disappearance of old ones ^16, 17^. In these three groups, selective therapeutic pressures can suppress responsive clones, allowing resistant clones to become dominant. Persons with high-risk disease are more likely to exhibit linear evolution or clonal tides. Clones with inherent genomic instability, detected at low levels at diagnosis, may undergo further genomic shifts when exposed to DNA-damaging agents, emerging after multiple relapses as the fatal clone. Understanding the clonal evolution pattern will become increasingly relevant as therapeutic choices expand in order to select appropriate treatment for patients. Re-emergence of a previously sensitive sub-clone would warrant the use of the same combination therapy at relapse. Alternatively, the emergence under treatment-induced selection pressure of a newly dominant linearly evolved or pre-existing resistant sub-clone should lead to alternate drugs.

## Newer proteasome inhibitors

Two new proteasome inhibitors (PIs) have been approved in recent years: carfilzomib (K) and ixazomib (I). Carfilzomib is an intravenous, irreversible PI with impressive outcomes in both newly diagnosed MM and relapsed MM. In patients with early relapse (one to three prior regimens), when compared with lenalidomide and dexamethasone (RD), carfilzomib in combination with lenalidomide and dexamethasone (KRD) improved PFS (hazard ratio (HR) 0.69, *P* = 0.0001) and OS (HR 0.79, *P* = 0.04) ^18^. In newly diagnosed patients, the KRD triplet had a response rate of 98%, a complete response rate of 56%, and MRD negativity rate by next-generation sequencing of 58%, and no discontinuation due to adverse events ^11^. Benefits of carfilzomib include rapid time to response, excellent tolerability (15% discontinued due to adverse events), efficacy in high-risk disease, and the low rate of carfilzomib-induced peripheral neuropathy. Challenges with carfilzomib include twice-weekly intravenous administration, the risk for tumor lysis syndrome, and the possibility for cardiac events and dyspnea. One retrospective study identified that more than 20% of patients developed systolic heart failure or arrhythmias. Previous irradiation to the thoracic spine and concomitant administration of doxorubicin increased the risk for cardiac dysfunction ^19^. To overcome these challenges, recent trials have demonstrated tolerability and response with escalated doses ^20^, whereas others are investigating efficacy of once-weekly dosing.

Ixazomib is an orally bioavailable, reversible PI with higher tissue distribution and a shorter dissociation half-life than bortezomib ^21^. It has modest single-agent activity and response to bortezomib-exposed persons when combined with dexamethasone ^22^. The combination of ixazomib with lenalidomide and dexamethasone (IRD) compared with RD shortened the time to response, improved the overall response rate, and increased median PFS (20.6 versus 14.7 months, HR 0.74, *P* = 0.012) ^23^. Early signal also suggests that it may overcome the negative impact of cytogenetic alterations. Major toxicities include gastrointestinal (GI) toxicity, peripheral neuropathy, and cyclical thrombocytopenia. The tissue distribution and dissociation half-life have a theoretical benefit with extramedullary disease, although this has not yet been demonstrated in a clinical trial. Ixazomib can be used as the proteasome backbone for combination oral therapies, reducing the need for frequent infusion visits. IRD is being investigated in newly diagnosed, transplant-ineligible persons with MM in a multi-center clinical trial, but results are not yet available. The availability of an oral PI also increases the feasibility of oral PI-based maintenance/consolidation regimens after transplant. Although it has been approved by the FDA, European regulators are waiting for additional positive data.

## Immunomodulatory drug

Immunomodulatory drug (IMID) can bind cereblon on the E3 ubiquitin ligase complex, enhancing the ubiquitination and degradation of two zinc finger transcription factors ^24, 25^. Pomalidomide is an oral third-generation IMID approved for relapsed refractory MM after at least two prior regimens. In lenalidomide-refractory patients, including 75% who were dual refractory, the response rate for pomalidomide and weekly dexamethasone was 30% with a 4-month PFS ^26^. In high-risk cytogenetics, patients with del(17p) had higher response rates, duration of response, and time to progression than those with t(4;14) ^27^. Pomalidomide is more effective earlier in the disease course ^28^, although approximately 40% of bortezomib- and lenalidomide-refractory patients can have a prolonged PFS ^29^. Toxicities, including cytopenias, rash, and peripheral neuropathy, are unpredictable ^30^. Unlike prior IMIDs, pomalidomide causes less endothelial stress and may be associated with lower rates of venous thromboembolic events, although patients should be on prophylactic anti-platelet therapy or anticoagulation ^31^. Its limited toxicity allows for combination with PIs, cyclophosphamide, and monoclonal antibodies, improving its efficacy.

## Histone deacetylase inhibitor

Panobinostat, a pan-histone deacetylase inhibitor (HDAC), targets HDAC enzymes involved in the aggresome protein degradation pathway, working synergistically with PIs. In a heavily pre-treated patient population, including 73% who were bortezomib-refractory, the response rate of panobinostat, bortezomib, and dexamethasone was 35% with a 5.4-month PFS ^32^. In bortezomib-refractory patients, the clinical benefit rate was better than 50% with a 4.2-month PFS. As high rates of GI distress and thrombocytopenia are seen with this agent, tolerability is a major impediment to its use and requires intense clinic/nursing support for continuous use. Dose interruptions occur in more than 50%, and relative dose intensity is only 73%. In a less heavily pre-treated population, response rates and PFS are improved, but the difficulty in maintaining dose intensity persists ^33^. Nonetheless, panobinostat has a novel mechanism of action, is an oral agent, and continues to have an important role in the management of MM, especially in the PI-refractory setting.

## Monoclonal antibody

The FDA has recently approved two monoclonal antibodies in relapsed or refractory MM (RRMM) or both—elotuzumab and daratumumab—which target the signaling lymphocytic activation molecule F7 (SLAMF7) and CD38, respectively ^34, 35^. In a phase III trial of patients with RRMM, the addition of elotuzumab to RD significantly improved PFS (19.4 versus 14.9 months, *P* < 0.001) ^35^. In addition, it improves the PFS of the subsequent therapy, suggesting a lasting effect on natural killer (NK) cells. Single-agent daratumumab produced a response rate of approximately 30% in heavily pre-treated patients and little toxicity other than infusion reactions ^34^. Another CD38 antibody, isatuximab (SAR650984), has shown similar single-agent activity in RRMM but is not commercially available ^36^. Both elotuzumab and daratumumab are being investigated in the front-line setting, and given the unprecedented single-agent activity, it is likely that daratumumab might be the backbone of MM treatment, akin to rituximab in B-cell lymphomas. Emerging data point to impressive responses for anti-CD38 antibodies in combination with IMIDs and PIs.

## Triplets at induction

When compared with doublets, triplet induction therapy containing a PI and an IMID results in a higher response rate and improved PFS and is preferred whenever possible in fit patients, especially given the synergism between these two drug classes ^37, 38^. A recent study has shown that bortezomib-lenalidomide and dexamethasone (VRD) has superior OS compared with RD ^39^. Bortezomib-thalidomide and dexamethasone (VTD) has shown superior response rates than thalidomide and dexamethasone (TD) and bortezomib-dexamethasone in randomized trials ^37^. Bortezomib-cyclophosphamide and dexamethasone (VCD) was recently shown to have lower response rates than VTD ^40^ ( Table 1). Although greater hematologic toxicity is seen in the former and peripheral neuropathy in the latter, the overall rate of grade 3/4 toxicity is similar, and individualization of therapy should dictate treatment decisions ^41^. The second-generation PI carfilzomib in combination with lenalidomide and dexamethasone (KRD) has also shown excellent response rates in phase II trials ^42^, but additional data and regulatory approval are necessary prior to broader use. Conversely, lenalidomide-based triplets failed to show a PFS or OS benefit over lenalidomide-based doublets in an elderly population ^43^. When compared with doublets, triplet therapies are associated with increased toxicity and usually longer PFS but not necessarily OS. The upfront use of the most active agents may limit treatment choices in future lines of therapy, possibly shortening the second and subsequent PFS periods. Given the prolonged survival in patients with myeloma as well as rapidly changing treatment modalities, this debate may be difficult to resolve.

**Table 1.** Triplet induction regimens and autologous stem cell transplantation studies in newly diagnosed multiple myeloma.

Regimen	Number of patients	Overall response rate	PFS	OS	Comments
VTD vs. TD ^38^	241 vs. 239	31% vs. 11%	NR	NR	NR
VTD vs. VCD ^42^	170 vs. 170	92.3% vs. 83.4%	NR	NR	NR
VRD vs. RD ^40^	242 vs. 232	71% vs. 63%	43 vs. 31 months	NR vs. 63 months	Median OS/PFS
Conventional chemo vs. ASCT ^48^	100 vs. 100	81% vs. 22%	28% vs. 10%	52% vs. 12%	Pre-novel era; 5-year OS/PFS
ASCT vs. MPR ^57^	141 vs. 132	NR	54.7 vs. 37.4 months	78.6% vs. 66.6%	Novel era PFS (median) OS (5-year)

ASCT, autologous stem cell transplantation; MPR, melphalan/prednisone and revlimid; NR, not reported; OS, overall survival; PFS, progression-free survival; RD, lenalidomide and dexamethasone; TD, thalidomide and dexamethasone; VCD, bortezomib-cyclophosphamide and dexamethasone; VRD, bortezomib-lenalidomide and dexamethasone; VTD, bortezomib-thalidomide and dexamethasone.

## Triplets at first relapse

Management of RRMM remains controversial, although the field is moving toward combination triplet therapy at first relapse in fit patients. In the US, an abundance of second-line therapies are available, allowing individualization of therapy to both the patient and the disease. In older persons with RRMM, a geriatric assessment should be performed to determine a frailty score, evaluating the Katz Activities of Daily Living, the Lawton Instrumental Activity of Daily Living, and the Charlson Comorbidity Index ^44^. Frail patients have shorter OS irrespectively of staging, treatment types, or physician-assessed performance status. Even when adjusted for International Staging System and chromosomal abnormalities, frail patients have shorter OS and PFS and higher rates of non-hematologic adverse events and treatment discontinuation and are likely to benefit from dose-adjusted therapy. Other validated frailty indexes evaluate similar factors as well as cytogenetics, renal function, and Karnofsky Performance Status ^45^.

The addition of a third drug to an IMID steroid combination improves response rates and PFS, although an OS benefit is not demonstrated consistently ^18, 35, 46^. The addition of bortezomib (intravenous) to TD prolongs time to progression (HR 0.59, *P* = 0.001) in previously transplanted patients and results in a trend toward improved 24-month survival (71% versus 65%, *P* = 0.093) ^46^. However, this population is not typical for the US, since only 20% were previously treated with bortezomib and 8% with thalidomide. Although VTD may have a limited role in the US, this study does illustrate the benefit of triplet therapy. The addition of carfilzomib to lenalidomide and dexamethasone improves not only response rates and PFS (HR 0.069, *P* = 0.0001) but also 24-month survival (HR 0.79, *P* = 0.04) ^11^. The data from other recent trials of triplets in early relapsed disease, though clearly demonstrating PFS benefit, did not show an OS advantage.

## Which triplet to use

The choice of treatment at relapse is determined by the type, response, and duration of initial therapy as well as disease factors, patient factors, and patient preference. The ideal sequence of therapies is not known, but switching drug classes is likely beneficial, especially in higher-risk disease, given the evidence for clonal tides. For instance, if there is disease progression while on lenalidomide maintenance, a triplet containing lenalidomide would likely provide a suboptimal response. If available, carfilzomib/IMID triplet should be considered in aggressive relapses, given the excellent data with rapid induction of response and high rates of MRD negativity. Those who relapse following a short duration of remission after upfront therapy, including autologous stem cell transplant, or symptomatic relapses with significant clinical symptoms or disease relapse in high-risk cytogenetics are generally considered to be aggressive relapses. Elotuzumab-based treatment could be considered in individuals who are frail or have underlying cytopenias. An ixazomib-based regimen is an option if an oral regimen is desired and, based on early trends, may be considered, especially in del(17p) or extramedullary disease. A pomalidomide-based regimen could also be considered in del(17p). Panobinostat has a limited role in first relapse because of the toxicity profile unless used to restore PI sensitivity in those relapsing after prior PI regimens.

## Transplant still better

High-dose therapy followed by autologous stem cell transplantation (ASCT) was the standard of care for transplant-eligible patients before the era of novel agents. In pre-novel agent randomized trials, ASCT was uniformly associated with improved response rates and PFS, and about half of the studies showed an OS benefit and superior quality of life ^47, 48^. In one of the studies designed to address the question of early versus late ASCT ^49^, patients undergoing ASCT were found to have longer time without symptoms and treatment, and thus the patients with ASCT were found to have improved quality of life.

Given the unprecedented response rates with novel agents, the role of ASCT as front-line therapy has been questioned ^50– 52^. Novel agents have significantly improved the rate of complete response and MRD, both surrogates for improved survival ^53, 54^. The early versus late ASCT was investigated in a number of randomized trials ^55, 56^, most recently in an international study conducted in the context of modern therapy with VRD induction, ASCT, and lenalidomide maintenance ^57^. Superior OS benefit was seen in one study ^56^ whereas in the other two studies, early ASCT was found to have better PFS with no transplant-related deaths ^55, 57^. Maintenance therapy after ASCT is gaining increased acceptance. Two randomized trials demonstrated a PFS benefit to lenalidomide maintenance, but only the Cancer and Leukemia Group B (CALGB) trial demonstrated an OS benefit ^58, 59^. The difference in results could be explained by the use of two cycles of consolidation chemotherapy after ASCT and only one year of maintenance lenalidomide in the IFM (Intergroupe Francophone du Myelome) trial. In addition, the majority of subjects in the CALGB study, unlike the IFM trial, received a lenalidomide-based induction regimen. In addition, an unplanned subset analysis in the CALGB trial failed to show an OS benefit in those who did not receive a lenalidomide-based induction regimen. Likewise, bortezomib maintenance can improve PFS and possibly OS ^60^. Further support for maintenance comes from the observation that maintenance lenalidomide, despite ASCT and consolidation therapy, can further deepen response, when measuring for MRD by flow ^61^. Last, newer data demonstrate the benefit to continuous therapy. A meta-analysis of three trials, one of which included ASCT as part of consolidation, demonstrated improved PFS1, PFS2 (time to second progression of death), and OS with continuous therapy. In fact, a prolonged PFS1 did not reduce PFS2 ^62^. In conclusion, novel agent-based triplet induction, front-line early ASCT, and maintenance therapy remain the preferred strategy for transplant-eligible patients.

## Immunotherapy

The best-established immunotherapy in MM is allogeneic transplantation with a potent and often sustained graft-versus-myeloma (GVM) effect ^63^. A series of prospective randomized studies, both in the US and Europe, explored the role of a tandem ASCT and reduced-intensity conditioning allogeneic stem cell transplants from a matched sibling or unrelated donor ^64, 65^. The results of these studies have been discordant, especially in the front-line treatment of MM ^65– 68^. Nonetheless, younger eligible patients with well-defined high-risk features should be considered for allogeneic transplant, especially within a clinical trial and early in the clinical course (front-line or first relapse). The risks of chronic graft-versus-host disease (cGVHD) and the need for long-term immunosuppression remain major challenges. A number of studies suggest that immune therapy works in MM and these include demonstration of clinically meaningful GVM effect by donor lymphocyte infusions in patients relapsing after allogeneic transplant ^69^, complete remission after immunosuppression withdrawal ^62^, progression after allogeneic transplant, and the presence of cGVHD correlating with freedom from progression after allogeneic transplant ^64^.

A variety of novel post-ASCT or allogeneic transplant immunotherapeutic strategies are now being explored in MM. These include cellular approaches such as myeloma-specific T cells (via T-cell expansion), marrow-infiltrating lymphocytes ^70^, redirected T cells with chimeric antigen receptors ^71, 72^, and tumor-based vaccines to induce myeloma-specific immunity in the context of enhanced antigen presentation ^73^. A phase I/II study showed that adoptive transfer of tumor antigen vaccine-primed and co-stimulated T cells leads to augmented and accelerated cellular and humoral immune reconstitution, including antitumor immunity, after ASCT ^74^. In another phase II trial, idiotype-pulsed antigen-presenting cells immunotherapeutic called APC8020 (Mylovenge), which was given after ASCT for MM, appeared to be associated with improved OS ^75^. In a recent report, deep sustained response to anti-CD19 chimeric antigen receptor T cells was reported in a patient with relapsed/refractory disease ^76^. Several promising antigenic targets have been identified for the development of anti-MM chimeric antigen receptors, such as B-cell maturation antigen, CD138, kappa light chains, and CS-1. Inhibitors of PD-1 and PDL-1 are being studied as a means of breaking down MM immune tolerance ^71, 77^. Though the anti-PD1 agent nivolumab was unimpressive ^78^, promising response rates were observed in combination with lenalidomide ^79^. NK cell therapy has generated interest in the treatment of MM and a number of studies are exploring this option. These include modulation of NK activity using anti-KIR Ab IPH2101 (monoclonal antibody against inhibitory KIR on NK cells) to establish MM-specific immunity ^80^ safety and efficacy study of autologous-, allogeneic-, and cord-derived NK cells ^81, 82^. A clinical trial involving the haploidentical allo-stem cell transplant followed by planned NK cell infusion attempts to use donor-recipient KIR ligand mismatch and NK cell reactivity to facilitate long-term remission (NCT02100891). Another promising area is the use of tumor vaccine based on the study in which patients receiving a patient-specific dendritic cell/myeloma fusion vaccine demonstrated the expansion of MM-specific T cells as well as upgrading of response in a subgroup of patients ^73^. A randomized phase II trial (BMT CTN 1401) with ASCT followed by lenalidomide maintenance with or without vaccination using the dendritic cell/myeloma fusion vaccine is currently accruing patients.

Hematopoietic cell transplantation provides an ideal platform for additional immune-based therapies. The recovery phase from ASCT (or other lymphodepleting therapy) represents a favorable platform for adoptive cellular therapy. The homeostatic lymphocyte proliferation following lymphopenia is a context in which immune checkpoint blockers may also be able to reverse MM-associated T-cell exhaustion ^71, 79^. Additionally, lymphopenia resulting from ASCT facilitates elimination of tolerogenic antigen-presenting cells and induces cytokine release that generates a more favorable environment for adoptive T-cell therapy. Indirect evidence suggests that the immune system can contribute to the clinical benefits of ASCT (for example, those with early lymphoid recovery after ASCT have superior long-term outcomes) ^83^.

## Conclusions

Although MM has always been considered incurable, many investigators now believe that a significant fraction of patients currently undergoing therapy may be cured ^84^. It is clear that with the improved understanding of disease biology and clonal architecture of relapse, combined with the availability of multi-targeted approaches, we are closer to a lasting cure than ever before. Nonetheless, many questions remain unanswered. Will the better understanding of myeloma biology guide the intensity of treatment, with high-risk disease necessitating aggressive therapy? With an increasing number of drug classes as well as new drugs in each class, the combination and sequencing of drugs are largely unresolved.

## References

[1] Howlader N Noone AM Krapcho M: SEER Cancer Statistics Review (CSR) 1975–2013. National Cancer Institute. Bethesda, MD; 4/2016 Reference Source

[2] Rajkumar SV Dimopoulos MA Palumbo A: International Myeloma Working Group updated criteria for the diagnosis of multiple myeloma. *Lancet Oncol.* 2014;15(12):e538–48. 10.1016/S1470-2045(14)70442-5 25439696

[3] Rajkumar SV Landgren O Mateos MV: Smoldering multiple myeloma. *Blood.* 2015;125(20):3069–75. 10.1182/blood-2014-09-568899 25838344 PMC4432003

[4] Rajkumar SV Gupta V Fonseca R: Impact of primary molecular cytogenetic abnormalities and risk of progression in smoldering multiple myeloma. *Leukemia.* 2013;27(8):1738–44. 10.1038/leu.2013.86 23515097 PMC3773463

[5] Neben K Jauch A Hielscher T: Progression in smoldering myeloma is independently determined by the chromosomal abnormalities del(17p), t(4;14), gain 1q, hyperdiploidy, and tumor load. *J Clin Oncol.* 2013;31(34):4325–32. 10.1200/JCO.2012.48.4923 24145347

[6] Kyle RA Remstein ED Therneau TM: Clinical course and prognosis of smoldering (asymptomatic) multiple myeloma. *N Engl J Med.* 2007;356(25):2582–90. 10.1056/NEJMoa070389 17582068

[7] Hillengass J Fechtner K Weber MA: Prognostic significance of focal lesions in whole-body magnetic resonance imaging in patients with asymptomatic multiple myeloma. *J Clin Oncol.* 2010;28(9):1606–10. 10.1200/JCO.2009.25.5356 20177023

[8] Dispenzieri A Kyle RA Katzmann JA: Immunoglobulin free light chain ratio is an independent risk factor for progression of smoldering (asymptomatic) multiple myeloma. *Blood.* 2008;111(2):785–9. 10.1182/blood-2007-08-108357 17942755 PMC2200851

[9] González-Calle V Dávila J Escalante F: Bence Jones proteinuria in smoldering multiple myeloma as a predictor marker of progression to symptomatic multiple myeloma. *Leukemia.* 2016. 10.1038/leu.2016.123 27133826

[10] Mateos MV Hernández MT Giraldo P: Lenalidomide plus dexamethasone for high-risk smoldering multiple myeloma. *N Engl J Med.* 2013;369(5):438–47. 10.1056/NEJMoa1300439 23902483

[11] Korde N Roschewski M Zingone A: Treatment With Carfilzomib-Lenalidomide-Dexamethasone With Lenalidomide Extension in Patients With Smoldering or Newly Diagnosed Multiple Myeloma. *JAMA Oncol.* 2015;1(6):746–54. 10.1001/jamaoncol.2015.2010 26181891 PMC6662597

[12] Palumbo A Avet-Loiseau H Oliva S: Revised International Staging System for Multiple Myeloma: A Report From International Myeloma Working Group. *J Clin Oncol. * 2015;33(26):2863–9. 10.1200/JCO.2015.61.2267 26240224 PMC4846284

[13] Morgan GJ Walker BA Davies FE: The genetic architecture of multiple myeloma. *Nat Rev Cancer.* 2012;12(5):335–48. 10.1038/nrc3257 22495321

[14] Bianchi G Munshi NC: Pathogenesis beyond the cancer clone(s) in multiple myeloma. *Blood.* 2015;125(20):3049–58. 10.1182/blood-2014-11-568881 25838343 PMC4432002

[15] Walker BA Boyle EM Wardell CP: Mutational Spectrum, Copy Number Changes, and Outcome: Results of a Sequencing Study of Patients With Newly Diagnosed Myeloma. *J Clin Oncol.* 2015;33(33):3911–20. 10.1200/JCO.2014.59.1503 26282654 PMC6485456

[16] Bolli N Avet-Loiseau H Wedge DC: Heterogeneity of genomic evolution and mutational profiles in multiple myeloma. *Nat Commun.* 2014;5:2997. 10.1038/ncomms3997 24429703 PMC3905727

[17] Keats JJ Chesi M Egan JB: Clonal competition with alternating dominance in multiple myeloma. *Blood.* 2012;120(5):1067–76. 10.1182/blood-2012-01-405985 22498740 PMC3412330

[18] Stewart AK Rajkumar SV Dimopoulos MA: Carfilzomib, lenalidomide, and dexamethasone for relapsed multiple myeloma. *N Engl J Med.* 2015;372(2):142–52. 10.1056/NEJMoa1411321 25482145

[19] Danhof S Schreder M Rasche L: ‘Real-life’ experience of preapproval carfilzomib-based therapy in myeloma - analysis of cardiac toxicity and predisposing factors. *Eur J Haematol.* 2016;97(1):25–32. 10.1111/ejh.12677 26331915

[20] Lendvai N Hilden P Devlin S: A phase 2 single-center study of carfilzomib 56 mg/m ^2^ with or without low-dose dexamethasone in relapsed multiple myeloma. *Blood.* 2014;124(6):899–906. 10.1182/blood-2014-02-556308 24963043 PMC4624439

[21] Kupperman E Lee EC Cao Y: Evaluation of the proteasome inhibitor MLN9708 in preclinical models of human cancer. *Cancer Res.* 2010;70(5):1970–80. 10.1158/0008-5472.CAN-09-2766 20160034

[22] Kumar SK LaPlant B Roy V: Phase 2 trial of ixazomib in patients with relapsed multiple myeloma not refractory to bortezomib. *Blood Cancer J.* 2015;5:e338. 10.1038/bcj.2015.60 26275080 PMC4558585

[23] Moreau P Masszi T Grzasko N: Oral Ixazomib, Lenalidomide, and Dexamethasone for Multiple Myeloma. *N Engl J Med.* 2016;374(17):1621–34. 10.1056/NEJMoa1516282 27119237

[24] Lu G Middleton RE Sun H: The myeloma drug lenalidomide promotes the cereblon-dependent destruction of Ikaros proteins. *Science.* 2014;343(6168):305–9. 10.1126/science.1244917 24292623 PMC4070318

[25] Krönke J Udeshi ND Narla A: Lenalidomide causes selective degradation of IKZF1 and IKZF3 in multiple myeloma cells. *Science.* 2014;343(6168):301–5. 10.1126/science.1244851 24292625 PMC4077049

[26] San Miguel J Weisel K Moreau P: Pomalidomide plus low-dose dexamethasone versus high-dose dexamethasone alone for patients with relapsed and refractory multiple myeloma (MM-003): a randomised, open-label, phase 3 trial. *Lancet Oncol.* 2013;14(11):1055–66. 10.1016/S1470-2045(13)70380-2 24007748

[27] Leleu X Karlin L Macro M: Pomalidomide plus low-dose dexamethasone in multiple myeloma with deletion 17p and/or translocation (4;14): IFM 2010-02 trial results. *Blood.* 2015;125(9):1411–7. 10.1182/blood-2014-11-612069 25575538

[28] Morgan G Palumbo A Dhanasiri S: Overall survival of relapsed and refractory multiple myeloma patients after adjusting for crossover in the MM-003 trial for pomalidomide plus low-dose dexamethasone. *Br J Haematol.* 2015;168(6):820–3. 10.1111/bjh.13227 25403264

[29] Fouquet G Pegourie B Macro M: Safe and prolonged survival with long-term exposure to pomalidomide in relapsed/refractory myeloma. *Ann Oncol.* 2016;27(5):902–7. 10.1093/annonc/mdw017 26787238

[30] Dimopoulos MA Leleu X Palumbo A: Expert panel consensus statement on the optimal use of pomalidomide in relapsed and refractory multiple myeloma. *Leukemia.* 2014;28(8):1573–85. 10.1038/leu.2014.60 24496300 PMC4131249

[31] Rosovsky R Hong F Tocco D: Endothelial stress products and coagulation markers in patients with multiple myeloma treated with lenalidomide plus dexamethasone: an observational study. *Br J Haematol.* 2013;160(3):351–8. 10.1111/bjh.12152 23240658 PMC4114520

[32] Richardson PG Schlossman RL Alsina M: PANORAMA 2: panobinostat in combination with bortezomib and dexamethasone in patients with relapsed and bortezomib-refractory myeloma. *Blood.* 2013;122(14):2331–7. 10.1182/blood-2013-01-481325 23950178

[33] San-Miguel JF Hungria VT Yoon SS: Panobinostat plus bortezomib and dexamethasone versus placebo plus bortezomib and dexamethasone in patients with relapsed or relapsed and refractory multiple myeloma: a multicentre, randomised, double-blind phase 3 trial. *Lancet Oncol.* 2014;15(11):1195–206. 10.1016/S1470-2045(14)70440-1 25242045

[34] Lokhorst HM Plesner T Laubach JP: Targeting CD38 with Daratumumab Monotherapy in Multiple Myeloma. *N Engl J Med.* 2015;373(13):1207–19. 10.1056/NEJMoa1506348 26308596

[35] Lonial S Dimopoulos M Palumbo A: Elotuzumab Therapy for Relapsed or Refractory Multiple Myeloma. *N Engl J Med.* 2015;373(7):621–31. 10.1056/NEJMoa1505654 26035255

[36] Martin T Richter J Vij R: A Dose Finding Phase II Trial of Isatuximab (SAR650984, Anti-CD38 mAB) As a Single Agent in Relapsed/Refractory Multiple Myeloma. *Blood.*ASH,2015;126(23):509 Reference Source

[37] Cavo M Tacchetti P Patriarca F: Bortezomib with thalidomide plus dexamethasone compared with thalidomide plus dexamethasone as induction therapy before, and consolidation therapy after, double autologous stem-cell transplantation in newly diagnosed multiple myeloma: a randomised phase 3 study. *Lancet.* 2010;376(9758):2075–85. 10.1016/S0140-6736(10)61424-9 21146205

[38] Moreau P Avet-Loiseau H Facon T: Bortezomib plus dexamethasone versus reduced-dose bortezomib, thalidomide plus dexamethasone as induction treatment before autologous stem cell transplantation in newly diagnosed multiple myeloma. *Blood.* 2011;118(22):5752–8; quiz 5982. 10.1182/blood-2011-05-355081 21849487

[39] Durie B Hoering A Rajkumar SV: Bortezomib, Lenalidomide and Dexamethasone vs. Lenalidomide and Dexamethasone in Patients (Pts) with Previously Untreated Multiple Myeloma Without an Intent for Immediate Autologous Stem Cell Transplant (ASCT): Results of the Randomized Phase III Trial SWOG S0777. *Blood.*ASH Annual Meeting Abstracts,2015;126(23):25 Reference Source 10.1038/s41408-020-0311-8 PMC7214419 32393732

[40] Reeder CB Reece DE Kukreti V: Cyclophosphamide, bortezomib and dexamethasone induction for newly diagnosed multiple myeloma: high response rates in a phase II clinical trial. *Leukemia.* 2009;23(7):1337–41. 10.1038/leu.2009.26 19225538 PMC2711213

[41] Moreau P Hulin C Macro M: VTD is superior to VCD prior to intensive therapy in multiple myeloma: results of the prospective IFM2013-04 trial. *Blood.* 2016;127(21):2569–74. 10.1182/blood-2016-01-693580 27002117

[42] Jakubowiak AJ Dytfeld D Griffith KA: A phase 1/2 study of carfilzomib in combination with lenalidomide and low-dose dexamethasone as a frontline treatment for multiple myeloma. *Blood.* 2012;120(9):1801–9. 10.1182/blood-2012-04-422683 22665938 PMC5162553

[43] Magarotto V Bringhen S Offidani M: Triplet vs doublet lenalidomide-containing regimens for the treatment of elderly patients with newly diagnosed multiple myeloma. *Blood.* 2016;127(9):1102–8. 10.1182/blood-2015-08-662627 26729895

[44] Palumbo A Bringhen S Mateos MV: Geriatric assessment predicts survival and toxicities in elderly myeloma patients: an International Myeloma Working Group report. *Blood.* 2015;125(13):2068–74. 10.1182/blood-2014-12-615187 25628469 PMC4375104

[45] Kleber M Ihorst G Terhorst M: Comorbidity as a prognostic variable in multiple myeloma: comparative evaluation of common comorbidity scores and use of a novel MM-comorbidity score. *Blood Cancer J.* 2011;1(9):e35. 10.1038/bcj.2011.34 22829196 PMC3255252

[46] Garderet L Iacobelli S Moreau P: Superiority of the triple combination of bortezomib-thalidomide-dexamethasone over the dual combination of thalidomide-dexamethasone in patients with multiple myeloma progressing or relapsing after autologous transplantation: the MMVAR/IFM 2005-04 Randomized Phase III Trial from the Chronic Leukemia Working Party of the European Group for Blood and Marrow Transplantation. *J Clin Oncol.* 2012;30(20):2475–82. 10.1200/JCO.2011.37.4918 22585692

[47] Attal M Harousseau JL Stoppa AM: A prospective, randomized trial of autologous bone marrow transplantation and chemotherapy in multiple myeloma. Intergroupe Français du Myélome. *N Engl J Med.* 1996;335(2):91–7. 10.1056/NEJM199607113350204 8649495

[48] Child JA Morgan GJ Davies FE: High-dose chemotherapy with hematopoietic stem-cell rescue for multiple myeloma. *N Engl J Med.* 2003;348(19):1875–83. 10.1056/NEJMoa022340 12736280

[49] Fermand JP Ravaud P Chevret S: High-dose therapy and autologous peripheral blood stem cell transplantation in multiple myeloma: up-front or rescue treatment? Results of a multicenter sequential randomized clinical trial. *Blood.* 1998;92(9):3131–6. 9787148

[50] Mellqvist UH Gimsing P Hjertner O: Bortezomib consolidation after autologous stem cell transplantation in multiple myeloma: a Nordic Myeloma Study Group randomized phase 3 trial. *Blood.* 2013;121(23):4647–54. 10.1182/blood-2012-11-464503 23616624 PMC3674665

[51] McCarthy PL Jr Hahn T Hassebroek A: Trends in use of and survival after autologous hematopoietic cell transplantation in North America, 1995–2005: significant improvement in survival for lymphoma and myeloma during a period of increasing recipient age. *Biol Blood Marrow Transplant.* 2013;19(17):1116–23. 10.1016/j.bbmt.2013.04.027 23660172 PMC3694566

[52] Cavo M Pantani L Petrucci MT: Bortezomib-thalidomide-dexamethasone is superior to thalidomide-dexamethasone as consolidation therapy after autologous hematopoietic stem cell transplantation in patients with newly diagnosed multiple myeloma. *Blood.* 2012;120(1):9–19. 10.1200/JCO.2009.23.7172 22498745

[53] Ladetto M Pagliano G Ferrero S: Major tumor shrinking and persistent molecular remissions after consolidation with bortezomib, thalidomide, and dexamethasone in patients with autografted myeloma. *J Clin Oncol.* 2010;28(12):2077–84. 10.1200/JCO.2009.23.7172 20308672

[54] Paiva B Vidriales M Cervero J: Multiparameter flow cytometric remission is the most relevant prognostic factor for multiple myeloma patients who undergo autologous stem cell transplantation. *Blood.* 2008;112(10):4017–23. 10.1182/blood-2008-05-159624 18669875 PMC2581991

[55] Gay F Oliva S Petrucci MT: Chemotherapy plus lenalidomide versus autologous transplantation, followed by lenalidomide plus prednisone versus lenalidomide maintenance, in patients with multiple myeloma: a randomised, multicentre, phase 3 trial. *Lancet Oncol.* 2015;16(16):1617–29. 10.1016/S1470-2045(15)00389-7 26596670

[56] Palumbo A Cavallo F Gay F: Autologous transplantation and maintenance therapy in multiple myeloma. *N Engl J Med.* 2014;371(10):895–905. 10.1056/NEJMoa1402888 25184862

[57] Attal M Lauwers-Cances V Hulin C: Autologous Transplantation for Multiple Myeloma in the Era of New Drugs: A Phase III Study of the Intergroupe Francophone Du Myelome (IFM/DFCI 2009 Trial). *Blood.* 2015 126(23):391 Reference Source

[58] McCarthy PL Owzar K Hofmeister CC: Lenalidomide after stem-cell transplantation for multiple myeloma. *N Engl J Med.* 2012;366(19):1770–81. 10.1056/NEJMoa1114083 22571201 PMC3744390

[59] Attal M Lauwers-Cances V Marit G: Lenalidomide maintenance after stem-cell transplantation for multiple myeloma. *N Engl J Med.* 2012;366(19):1782–91. 10.1056/NEJMoa1114138 22571202

[60] Rosiñol L Oriol A Teruel AI: Superiority of bortezomib, thalidomide, and dexamethasone (VTD) as induction pretransplantation therapy in multiple myeloma: a randomized phase 3 PETHEMA/GEM study. *Blood.* 2012;120(8):1589–96. 10.1182/blood-2012-02-408922 22791289

[61] Roussel M Lauwers-Cances V Robillard N: Front-line transplantation program with lenalidomide, bortezomib, and dexamethasone combination as induction and consolidation followed by lenalidomide maintenance in patients with multiple myeloma: a phase II study by the Intergroupe Francophone du Myélome. *J Clin Oncol.* 2014;32(25):2712–7. 10.1200/JCO.2013.54.8164 25024076

[62] Palumbo A Gay F Cavallo F: Continuous Therapy Versus Fixed Duration of Therapy in Patients With Newly Diagnosed Multiple Myeloma. *J Clin Oncol.* 2015;33(30):3459–66. 10.1200/JCO.2014.60.2466 26282661

[63] Tricot G Vesole DH Jagannath S: Graft-versus-myeloma effect: proof of principle. *Blood.* 1996;87(3):1196–8. 8562947

[64] Krishnan A Pasquini MC Logan B: Autologous haemopoietic stem-cell transplantation followed by allogeneic or autologous haemopoietic stem-cell transplantation in patients with multiple myeloma (BMT CTN 0102): a phase 3 biological assignment trial. *Lancet Oncol.* 2011;12(13):1195–203. 10.1016/S1470-2045(11)70243-1 21962393 PMC3611089

[65] Gahrton G Iacobelli S Bjorkstrand B: Autologous/reduced-intensity allogeneic stem cell transplantation vs autologous transplantation in multiple myeloma: long-term results of the EBMT-NMAM2000 study. *Blood.* 2013;121(25):5055–63. 10.1182/blood-2012-11-69452 23482933

[66] Giaccone L Storer B Patriarca F: Long-term follow-up of a comparison of nonmyeloablative allografting with autografting for newly diagnosed myeloma. *Blood.* 2011;117(24):6721–7. 10.1182/blood-2011-03-339945 21490341 PMC3251223

[67] Björkstrand B Iacobelli S Hegenbart U: Tandem autologous/reduced-intensity conditioning allogeneic stem-cell transplantation versus autologous transplantation in myeloma: long-term follow-up. *J Clin Oncol.* 2011;29(22):3016–22. 10.1200/JCO.2010.32.7312 21730266

[68] Bruno B Rotta M Patriarca F: Nonmyeloablative allografting for newly diagnosed multiple myeloma: the experience of the Gruppo Italiano Trapianti di Midollo. *Blood.* 2009;113(14):3375–82. 10.1182/blood-2008-07-167379 19064724 PMC2665903

[69] Lokhorst HM Schattenberg A Cornelissen JJ: Donor lymphocyte infusions for relapsed multiple myeloma after allogeneic stem-cell transplantation: predictive factors for response and long-term outcome. *J Clin Oncol.* 2000;18(16):3031–7. 10944138 10.1200/JCO.2000.18.16.3031

[70] Noonan KA Huff CA Davis J: Adoptive transfer of activated marrow-infiltrating lymphocytes induces measurable antitumor immunity in the bone marrow in multiple myeloma. *Sci Transl Med.* 2015;7(288):288ra78. 10.1126/scitranslmed.aaa7014 25995224 PMC4634889

[71] Rosenblatt J Glotzbecker B Mills H: PD-1 blockade by CT-011, anti-PD-1 antibody, enhances *ex vivo* T-cell responses to autologous dendritic cell/myeloma fusion vaccine. *J Immunother.* 2011;34(5):409–18. 10.1097/CJI.0b013e31821ca6ce 21577144 PMC3142955

[72] Garfall AL Fraietta JA Maus MV: Immunotherapy with chimeric antigen receptors for multiple myeloma. *Discov Med.* 2014;17(91):37–46. 24411699

[73] Rosenblatt J Vasir B Uhl L: Vaccination with dendritic cell/tumor fusion cells results in cellular and humoral antitumor immune responses in patients with multiple myeloma. *Blood.* 2011;117(2):393–402. 10.1182/blood-2010-04-277137 21030562 PMC3031474

[74] Rapoport AP Aqui NA Stadtmauer EA: Combination immunotherapy using adoptive T-cell transfer and tumor antigen vaccination on the basis of hTERT and survivin after ASCT for myeloma. *Blood.* 2011;117(3):788–97. 10.1182/blood-2010-08-299396 21030558 PMC3035073

[75] Lacy MQ Mandrekar S Dispenzieri A: Idiotype-pulsed antigen-presenting cells following autologous transplantation for multiple myeloma may be associated with prolonged survival. *Am J Hematol.* 2009;84(12):799–802. 10.1002/ajh.21560 19899131 PMC2908015

[76] Garfall AL Stadtmauer EA June CH: Chimeric Antigen Receptor T Cells in Myeloma. *N Engl J Med.* 2016;374(2):194. 10.1056/NEJMc1512760 26760100

[77] Benson DM Jr Bakan CE Mishra A: The PD-1/PD-L1 axis modulates the natural killer cell versus multiple myeloma effect: a therapeutic target for CT-011, a novel monoclonal anti-PD-1 antibody. *Blood.* 2010;116(13):2286–94. 10.1182/blood-2010-02-271874 20460501 PMC3490105

[78] Lekoshin AM Ansell SM Armand P: Preliminary Results of a Phase I Study of Nivolumab (BMS-936558) in Patients with Relapsed or Refractory Lymphoid Malignancies. *Blood.*ASH, Abstract,2014;124(21):291 Reference Source

[79] San Miguel J Mateos MV Shah JJ: Pembrolizumab in combination with Lenalidomide and low-dose dexamethasone for Relapsed/Refractory Multiple Myeloma (RRMM): Keynote-023. *Blood.*ASH,2015;126(23):505 Reference Source

[80] Benson DM Jr Hofmeister CC Padmanabhan S: A phase 1 trial of the anti-KIR antibody IPH2101 in patients with relapsed/refractory multiple myeloma. *Blood.* 2012;120(22):4324–33. 10.1182/blood-2012-06-438028 23033266 PMC3507143

[81] Shah N Li L Kaur I: Infusion of *Ex Vivo* Expanded Allogeneic Cord Blood-Derived Natural Killer Cells in Combination with Autologous Stem Cell Transplantation for Multiple Myeloma: Results of a Phase I Study. *Blood.*ASH,2015;126(23):929 Reference Source 26294715

[82] Szmania S Lapteva N Garg T: *Ex vivo*-expanded natural killer cells demonstrate robust proliferation *in vivo* in high-risk relapsed multiple myeloma patients. *J Immunother.* 2015;38(1):24–36. 10.1097/CJI.0000000000000059 25415285 PMC4352951

[83] Porrata LF Gertz MA Inwards DJ: Early lymphocyte recovery predicts superior survival after autologous hematopoietic stem cell transplantation in multiple myeloma or non-Hodgkin lymphoma. *Blood.* 2001;98(3):579–85. 10.1182/blood.V98.3.579 11468153

[84] Barlogie B Mitchell A van Rhee F: Curing myeloma at last: defining criteria and providing the evidence. *Blood.* 2014;124(20):3043–51. 10.1182/blood-2014-07-552059 25293776 PMC4231416

